# Factors influencing peripheral intravenous catheter practice of nurses in small and medium sized hospitals: a cross-sectional study

**DOI:** 10.1186/s12912-024-02026-4

**Published:** 2024-05-22

**Authors:** Jung Hee Kim, Inju Hwang, Eun Man Kim

**Affiliations:** 1 Department of Nursing Science, Shinsung University, Chungnam, South Korea, 1 Daehak-ro, Jeongmi-myeon, Dangjin-si, Chungnam, 31801 South Korea; 2 Department of Nursing Science, Sun Moon University, 70 Sunmoonro 221beongil, Tangjeongmyeon, Asansi, Chungnam, 31460 South Korea

**Keywords:** Peripheral catheterization, Nurses, Work environment, Patient safety

## Abstract

**Background:**

The importance of the peripheral intravenous catheter (PIVC) practices on patient safety is increasing. Small and medium-sized hospitals play a central role in the provision of healthcare services in South Korea, but lack a system for quality improvement, leaving patient safety at risk. This study aimed to identify the extent to which the PIVC practice knowledge of nurses, the nursing working environment, and the patient safety-culture perception affect PIVC practices and thereby provide basic data for improving the PIVC practices in small and medium-sized hospitals.

**Methods:**

This study had a cross-sectional descriptive design to identify the factors affecting PIVC nursing practices in small and medium-sized hospitals. Questionnaires returned by 149 nurses collected data on general characteristics, practical knowledge of PIVC nursing, nursing working environment, patient safety-culture perception, and PIVC practices. The questionnaire data were analysed using descriptive statistics, the independent *t*-test, one-way ANOVA, Scheffé’s test, Pearson correlation, and hierarchical regression analysis.

**Results:**

The mean score of PIVC practices was 4.60 out of 5. Length of clinical experience, practical knowledge of PIVC nursing and patient safety-culture perception were significant factors affecting the PIVC nursing practices, with these variables explaining 26.2% of the variance therein.

**Conclusions:**

The PIVC practices of nurses in small and medium-sized hospitals can be improved by providing education and training based on the latest standard or guideline to facilitate the acquisition of knowledge and skills. And campaigns and programs to strengthen patient safety culture perception specific to small and medium-sized hospital should be implemented. to ensure the safety of PIVC practice.

## Background

The peripheral intravenous catheter (PIVC) is the most-used invasive medical device in hospitals, and the importance of its effects on patient safety is increasing [1]. PIVC insertion is a procedure commonly performed by nurses that consumes a significant amount of nursing time [2]. Phlebitis is a common complication associated with the use of PIVCs that can lead to patient discomfort, vein damage, omission of medication, infectionor blood clot, ultimately leading to negative consequences of increased treatment costs and prolonged hospitalization [3].

Small and medium-sized hospitals, which account for about 93.8% of all medical institutions in Korea, play a large role in the supply of healthcare services as secondary medical institutions in the healthcare delivery system, and perform the central and core mission of healthcare services by providing convenience and accessibility to hospitals, especially for local residents (Korea Hospital Association, 2017) [4]. However small and medium-sized hospitals, which play a key role in healthcare services in South Korea, have become blind spots for patient safety issues due to the lack of nursing staff, lack of educational opportunities, communication problems, lack of interest in improving nurses’ performance and quality, and lack of personnel and systems for quality improvement.

Therefore identifying how nurses perform PIVC practices in small and medium- sized hospitals will provide the basis for improving the high-frequency, invasive procedures performed by nurses to ensure patient safety. However, there has been little research into how nurses perform the PIVC practices in small and medium-sized hospitals. Nurse competency for PIVC practices requires the acquisition of knowledge and skills across the continuum of pre-insertion, insertion and post-insertion [5]. Previous studies have confirmed gaps in nurses’ knowledge about managing and maintaining PIVC practices. Inadequate nursing knowledge related to routine treatment and maintenance of PIVC practices was found to be associated with poor patient outcomes, including higher complication, morbidity and mortality rates, and extended hospital stays [6–8]. A survey by the Infusion Nurses Society [9] revealed that most nurses were not formally trained in learning about the theoretical basis for PIVC insertion and management, instead primarily receiving on-site training. This has resulted in inadequate nurses having inadequate knowledge in areas such as the rational selection of the vascular access type and evidence-based PIVC management [9].

Organization-level variables that are predicted to influence PIVC nursing practices include the patient safety-culture perception and nursing working environment. The perception of the infection-control organizational culture among general hospital nurses is the most important predictor for whether healthcare-associated infection-control guidelines are implemented [10]. Meanwhile, the perception of infection-control organizational culture among emergency-department nurses is an important factor in the implementation of nursing practices for patient safety [11]. This situation makes it necessary to identify the effects of patient safety-culture perception and the nursing working environment on PIVC practices and to design and implement strategies to improve them. However, in small and medium-sized hospitals in South Korea, medical-related surveillance activities were only implemented after the Patient Safety Act of 2016, and infection-control facilities, personnel and equipment are often still inadequate [12]. Kim et al. (2013) [2] found that a lack of time and apprenticeship training were also influenced by situational factors and contribute to the low performance rate of PIVC practices in small and medium-sized hospitals. Therefore, improving these practices requires identification of the influencing factors among nurses working in small and medium-sized hospitals, who account for 60% of nurses in all medical institutions [13].

This study aimed to identify the extent to which the nurses’ knowledge of PIVC nursing, nursing working environment and patient safety culture perception affect PIVC nursing practices and thereby provide basic data for improving PIVC nursing practices in small and medium-sized hospitals.

The purposes of this study were as follows: (1) to identify the knowledge of PIVC nursing, PIVC nursing practices level, patient safety culture perception and nursing working environment among nurses in small and medium-sized hospitals, (2) to identify differences in PIVC practices according to the general characteristics of the subjects and (3) to identify factors that affect the PIVC nursing practices.

## Methods

### Design

A cross-sectional descriptive design adhering to the STROBE guidelines was employed to identify the factors affecting the PIVC nursing practices in small and medium-sized hospitals.

### Participants

A convenience sample of nurses practising intravenous practice in small and medium-sized hospitals in Chungcheongnam-do and Daejeon-do was conducted.

Inclusion criteria: (1) nurses from small and medium-sized hospitals with fewer than 400; (2) performing PIVC practices. Exclusion criteria: (1) clinical experience < 6 months; (2) nurses who do not directly care for patients working in outpatient, operating theatre or administrative support departments. In this study, we excluded new nurses with less than 6 months of experience to get a better understanding of the overall practice of nurses in small and medium-sized hospitals. The required number of participants was calculated using G*Power software (version 3.1). For a medium effect size of 0.15, a significance level of 0.05, a statistical power of 0.90 and 7 predictors, the target number of participants was 130. Therefore, 150 questionnaires were distributed to account for a 15% dropout rate, and all 149 returned questionnaires were used in the analysis.

### Instruments

#### Participant and PIVC nursing practice-related characteristics

This tool included gender, age, clinical experience, department of work, availability of an infection-control room and an infection-control nurse, availability of nurse educators, PIVC training status, PIVC practice education methods and frequency, presence of PIVC practice manual, PIVC practice guideline type, problems related to PIVC practices resolution method and PIVC nursing practice education necessity.

#### Practical knowledge of PIVC nursing

The researcher revised and supplemented the questionnaire based on the study of Choi & Jeong (2020) [14]. The content validity was verified by three field experts and two nursing professors, with the Content Validity Index (CVI) of 0.93.

The applied instrument consisted of six subscales: compliance with aseptic techniques, storage and handling, administering an injection, phlebitis assessment, interchange of intravenous tubes and fluid sets, and training for caregivers. The final 32 items were answered using “yes”, “no”, and “don’t know”, with each item scored as 1 point for a correct answer and 0 points for incorrect and don’t-know answers, where a higher score indicated a higher knowledge level of PIVC nursing. The reliability of the tool was indicated by Kuder-Richardson Formula 20(KR-20) of 0.410. This is similar to the value from the KR-20 in Choi & Jeong (2019) [14] of 0.46.

#### PIVC nursing practice

The researcher revised and supplemented the questionnaire based on the study of Choi & Jeong (2020) [14]. The content validity was verified by three field experts and two nursing professors, with the CVI of 0.94.

The applied instrument consisted of six subscales: compliance with aseptic techniques, storage and handling, administering an injection, phlebitis assessment, interchange of intravenous tubes and fluid sets, and training for caregivers. There were 24 questions in total, with question answered using a five-point Likert scale from 1 to 5, where a higher score indicated a higher administration level of PIVC practices. The reliability of the tool was indicated by a Cronbach’s α of 0.70, and it was 0.84 in the study of Choi & Jeong (2020) [14].

#### Nursing working environment

The nursing working environment was measured using the PES-NWI nursing working environment measurement instrument reported by Lake (2007) [15] and translated by Cho et al. (2011) [16]. The Korean version of the instrument has been validated for content validity and reliability, and consisted of five subscales: nurse participation in hospital affairs, nursing foundations for quality of care, nurse manager ability, leadership and support of nurses, staffing and resource adequacy, and collegial nurse–physician relations. The total number of items was 29, with each is scored on a five-point Likert scale, where higher scores indicated a better working environment for nurses. The reliability of the instrument at the time of development was indicated by a Cronbach’s α of 0.93, which in this study was 0.94.

#### Patient safety culture perception

The patient safety-culture perception was measured using the hospital patient safety culture developed by the Agency for Healthcare Research and Quality and adapted by Kim et al. (2007) [17]. The patient safety-culture perception tool consisted of five subscales: managers, communication and procedures, frequency of reporting incidents of medical errors, general safety of patients and interdepartmental cooperation. There were 37 questions in total, with each question scored on a five-point Likert scale, where higher scores indicated higher patient safety-culture perception. The reliability of the instrument at the time of development was indicated by a Cronbach’s α of 0.93, which in this study was 0.93.

### Data collection

Participants were recruited by visiting the nursing departments of four small and medium-sized hospitals in Chungcheongnam-do and Daejeon between 12th and 19th May and obtaining permission to conduct the study. Questionnaires were distributed to nurses who agreed to participate in the study between 22nd May and 15th June, and the completed questionnaires were collected by the nursing departments and returned by mail. It tooks 20 ~ 30 min to complete the questionnaire. It was explained to the study participants that they could withdraw from the study at any time. After the participants completed the survey, they were offered an e-coupon.

### Data analysis

The data collected in this study were analysed using SPSS software (version 23.0, IBM) as follows:

1. Analysis of the characteristics of the participants to obtain frequency, percentage and mean ± SD values.
2. The practical PIVC nursing knowledge and PIVC practices of participants were analysed to obtain mean ± SD values.
3. Differences between PIVC practices according to the characteristics of the participants were identified using the independent *t*-test, one-way ANOVA and post-hoc Scheffé’s test.
4. Pearson’s correlations were used to determine the relationships between PIVC nursing knowledge, nursing working environment, patient safety-culture perception and PIVC nursing practices.
5. Hierarchical regression analysis was conducted to determine the factors affecting PIVC nursing practices.

### Ethical consideration

This study collected data after deliberation by the SM University Institutional Review Board (Approval number: SM-202304-012-2, May 3. 2023).

## Results

### Participants characteristics

The characteristics of the participants are listed in Table 1. The participants included 53 (35.6%) with 1–5 years of clinical experience. An infection-control room and an infection-control nurse was present for 143 (96.0%) of the respondents, and 138 (92.6%) reported that they had a nurse educator. Most (*n* = 61, 40.9%) had received intravenous drug education within the past year, and the multiple-responses analysis of 61 respondents’ education methods revealed that 29 (47.5%) had received in-house lectures while 38 (43.7%) had received education periodically during their employment.

**Table 1:** Participant characteristics and PIVC practice-related characteristics (*n* = 149)

Characteristic	Categories	Frequency	Percentage
Gender	Male	8	5.4
	Female	141	94.6
Age, years	< 25	27	18.1
	25–29	41	27.5
	30–34	27	18.1
	35–39	23	15.5
	> 39	31	20.8
Length of clinical experience, year	< 1	21	14.1
	1–5	53	35.6
	6–10	26	17.4
	11–15	20	13.4
	> 15	29	19.5
Department	Internal medicine wards	36	24.2
	Surgical wards	23	15.4
	Special departments (e.g. ICU, emergency)	41	27.5
	Comprehensive nursing-care service units	34	22.8
	Others	15	10.1
Availability of an infection-control room and an infection-control nurse	Yes	143	96.0
	No	6	4.0
Availability of nurse educators	Yes	138	92.6
	No	11	7.4
PIVC training within the past year	Yes	61	40.9
	No	88	59.1
Methods of PIVC practice education*	Distribution of institutional training materials	19	27.6
	Lectures at their institutes	29	42.0
	Video material at their institutes	11	15.9
	External	7	10.1
	Others	3	4.4
Frequency of education on PIVC nursing practices	During the onboarding processes and periodically	38	43.7
	Annually	13	14.9
	Once while employed	7	8.1
	Once during the onboarding processes	29	33.3
Presence of PIVC practice manual	Yes	117	78.5
	No	32	21.5
Types of PIVC practice guideline*	Guidelines within their department	13	10.7
	Guidelines within the nursing department or infection-control room	77	63.1
	Standard guidelines established by the Hospital Nurses Association	29	23.8
	Others	3	2.4
How to resolve problems related to PIVC practices	Asked seniors or coworkers	106	71.1
	Solved them alone (e.g. referring to and searching for guidelines)	43	28.9
Need for PIVC nursing practice education	Necessary	109	73.2
	Unnecessary	40	26.8

* Multiple responses possible

According to the multiple-responses analysis of the types of guidelines for PIVC nursing practices among the 117 respondents, 77 (65.8%) had received guidelines related to infection control. Regarding how to resolve problems with PIVC nursing practices, 106 (71.1%) asked their seniors or coworkers, and 109 (73.2%) responded that they needed PIVC nursing practice training.

### Practical knowledge of PIVC nursing, PIVC practice, nursing working environment, and patient safety culture perception

The analysis results for the participants’ practical knowledge of PIVC nursing, PIVC nursing practices, nursing working environment and patient safety culture perception are presented in Table 2.

**Table 2:** Level of practical knowledge of PIVC nursing, PIVC practices, nursing working environment and patient safety-culture perception (*n* = 149)

Variables		Total		Average
		Range	Mean ± SD	Mean ± SD
Practical knowledge of PIVC nursing	Compliance with aseptic techniques	0–10	9.40 ± 0.74	0.94 ± 0.07
	Storage and handling	0–4	3.90 ± 0.32	0.97 ± 0.08
	Administering an injection	0–11	9.83 ± 0.94	0.89 ± 0.09
	Phlebitis assessment	0–2	1.82 ± 0.39	0.91 ± 0.19
	Interchange of intravenous tubes and fluid sets	0–3	1.84 ± 0.49	0.61 ± 0.16
	Training for caregivers	0–2	1.98 ± 0.14	0.99 ± 0.07
	Total	0–32	28.77 ± 1.69	0.90 ± 0.05
PIVC nursing practices	Compliance with aseptic techniques	8–40	36.81 ± 3.17	4.60 ± 0.40
	Storage and handling	3–15	14.18 ± 1.50	4.73 ± 0.50
	Administering an injection	7–35	29.33 ± 3.54	4.19 ± 0.51
	Phlebitis assessment	2–10	8.57 ± 1.76	4.29 ± 0.88
	Interchange of intravenous tubes and fluid sets	2–10	8.57 ± 1.71	4.29 ± 0.85
	Training for caregivers	2–10	9.53 ± 1.07	4.77 ± 0.53
	Total	24–120	106.98 ± 8.18	4.46 ± 0.34
Nursing working environment		29–116	80.06 ± 13.74	2.76 ± 0.47
Patient safety-culture perception	Managers	5–25	20.11 ± 3.29	4.02 ± 0.66
	Communication and procedures	13–65	47.97 ± 7.35	3.69 ± 0.57
	Frequency of reporting incidents of medical errors	4–20	14.89 ± 3.46	3.72 ± 0.87
	General safety of patients	1–5	3.76 ± 0.83	3.76 ± 0.83
	Interdepartmental cooperation	14–70	51.22 ± 8.67	3.66 ± 0.62
	Total	37–185	137.91 ± 18.75	3.73 ± 0.51

### Differences in PIVC practices according to the participants’ general characteristics

Table 3 presents the results of analysing the level of PIVC practices according to the characteristics of the participants. The characteristic that significantly affected the level of PIVC practices was the length of clinical experience (*F* = 2.72, *p* = .032), with this being better for 11–15 years of experience than for < 1 year of experience.

**Table 3:** Differences in PIVC practices according to the participants’ characteristics (*n* = 149)

Characteristic	Categories	Mean ± SD	t or F	*p* (Scheffé)
Gender	Male	4.37 ± 0.27	–0.73	0.467
	Female	4.46 ± 0.34		
Age, years	< 25^a^	4.44 ± 0.29	0.75	0.561
	25–29^b^	4.44 ± 0.34		
	30–34^c^	4.39 ± 0.38		
	35–39^d^	4.47 ± 0.29		
	> 39^e^	4.54 ± 0.38		
Length of clinical experience	< 1^a^	4.38 ± 0.33	2.72	0.032
	1–5^b^	4.43 ± 0.34		(d > a)
	6–10^c^	4.39 ± 0.37		
	11–15^d^	4.67 ± 0.25		
	> 15^e^	4.48 ± 0.35		
Department	Internal medicine wards	4.46 ± 0.34	0.99	0.416
	Surgical wards	4.34 ± 0.33		
	Special department	4.46 ± 0.31		
	Comprehensive nursing-care service units	4.52 ± 0.36		
	Others	4.46 ± 0.39		
Availability of an infection-control room and an infection-control nurse	Yes	4.46 ± 0.34	0.20	0.843
	No	4.43 ± 0.34		
Availability of nurse educators	Yes	4.46 ± 0.34	0.91	0.364
	No	4.37 ± 0.41		
PIVC training within the past year	Yes	4.50 ± 0.32	1.14	0.255
	No	4.43 ± 0.36		
Presence of PIVC practice manual	Yes	4.46 ± 0.35	0.37	0.716
	No	4.44 ± 0.31		
How to resolve questions related to PIVC practices	Asked seniors or coworkers	4.43 ± 0.35	–1.78	0.078
	Solved them alone (e.g. referring to and searching for guidelines)	4.53 ± 0.31		
Need for PIVC practice education	Necessary	4.46 ± 0.34	–0.04	0.970
	Unnecessary	4.46 ± 0.35		

### Relations between practical knowledge of PIVC nursing, nursing work environment, patient safety culture perception and PIVC practice

Table 4 presents the results of analysing the relationships between the participants’ practical knowledge of PIVC nursing, nursing working environment, patient safety culture perception and level of PIVC nursing practices.

**Table 4:** Relationships between practical knowledge of PIVC nursing, nursing working environment, patient safety-culture perception and PIVC practices (*n* = 149)

Variables	1	2	3	4
	*r*(*p*)			
1. Practical knowledge of PIVC nursing	1			
2. Nursing working environment	0.031 (0.711)	1		
3. Patient safety culture perception	0.014 (0.861)	0.630 (< 0.001)	1	
4. PIVC nursing practices	0.433 (< 0.001)	0.144 (0.080)	0.297 (< 0.001)	1

The level of PIVC nursing practices was significant correlated with practical knowledge of PIVC nursing (*r* = .433, *p* < .001) and patient safety culture perception (*r* = .297, *p* < .001). However, no significant correlation was observed for the nursing working environment (*r* = .144, *p* = .080).

### Factors influencing the PIVC practice

Hierarchical regression analysis was applied to identify the factors affecting the PIVC nursing practices; the results are presented in Table 5.

**Table 5:** Factors influencing the PIVC nursing practices (*n* = 149)

Variables	Model 1					Model 2				
	B	SE	β	t	*p*	B	SE	β	t	*p*
Constant	4.39	0.07		60.31	< 0.001	1.41	0.46		3.06	0.003
Clinical experience (**reference < 1 year**)										
1–5 years	0.04	0.09	0.06	0.47	0.639	0.00	0.08	0.00	0.01	0.995
5–10 years	0.01	0.10	0.01	0.08	0.937	0.03	0.09	0.03	0.34	0.736
10–15 years	0.29	0.10	0.29	2.76	0.007	0.15	0.10	0.15	1.57	0.119
> 15 years	0.10	0.10	0.11	1.00	0.322	0.04	0.09	0.04	0.40	0.687
Practical knowledge of PIVC nursing						2.57	0.48	0.40	5.30	< 0.001
Patient safety culture perception						0.19	0.05	0.28	3.87	< 0.001
Adjusted *R*^2^	0.044					0.262				
*R* ^2^	0.070					0.292				
*R*^2^ variation	-					0.221				
*F*(*p*)	2.72 (0.032)					9.74 (< 0.001)				
*F* variation (*p*)	-					22.19 (< 0.001)				

Durbin-Watson statistic = 1.776, tolerance = 0.419–0.940, VIF = 1.063–2.389

Before the analysis, the Durbin-Watson statistic indicated that there was no self-correlation between the errors, with a value of 1.795 (i.e. near to 2). The tolerance limit and the VIF (variance inflation factor) were both 1.000, indicating that there was no multicollinearity among the input independent variables.

Hierarchical regression analysis was conducted to examine the effect of PIVC practice knowledge and patient safety-culture perception on PIVC practice administration. Model 1 included work experience (< 1 year), which showed a significant difference in PIVC nursing practices among the surveyed characteristics, and Model 2 additionally included PIVC practice knowledge and patient safety-culture perception.

In Model 1, 11–15 years of clinical experience (*β* = 0.29, *p* = .007) was associated with a significantly higher level of PIVC practices than was < 1 year of clinical experience. The explanatory power of model 1 was 7.0% (*F* = 2.72, *p* = .032, *R*^2^ = 0.070, adjusted *R*^2^ = 0.044).

The additional inputs in Model 2 of PIVC nursing knowledge (*β* = 0.40, *p* < .001) and patient safety-culture perception (*β* = 0.28, *p* < .001) significantly affected PIVC practices, with higher PIVC practices associated with higher PIVC practice knowledge and higher patient safety-culture perception. The additional percentage explained by model 2 was 22.1%, and the total explanatory power of clinical experience in model 2 was 29.2% (*F* = 9.74, *p* < .001, *R*^2^ = 0.292, adjusted *R*^2^ = 0.262).

## Discussion

This study aimed to identify the extent of practical knowledge of PIVC nursing, nursing working environment, patient safety culture perception among nurses in small and medium-sized hospital nurses, and factors affecting PIVC nursing practices. The participants’ practical knowledge of PIVC nursing was 28.77 ± 1.69 out of a maximum possible score of 32 points. This is similar to the score of 25.63 ± 2.07 out of 29 points found by Choi & Jeong (2020) [14], who measured the level of practical knowledge of PIVC insertion among nurses in small and medium-sized hospitals. The score in the present study was higher than that of 16.01 ± 1.54 out of 22 points found by Song (2020) [18], who measured the level of practical knowledge of safe injections among nurses in small and medium-sized hospital. This difference is due to the increasing importance of infection control related to PIVC nursing practices for patient safety, with practice guidelines such as the Hospital Nurses Association (2017) [19] and the Ministry of Health and Welfare (2021) [20] now being available in wards and utilized actively. In particular, among the detailed items, there were high scores for compliance with aseptic procedures such as hand hygiene and disinfection of insertion sites. This is also believed to be due to the strengthening of infection-control education emphasizing hand hygiene and compliance with sterilization procedures. The item with the lowest score among the specific items of this study pertained to adherence to infusion time according to the characteristics of the infusion solution and the exchange of infusion sets. Therefore, additional education and verification of practices regarding infusion time based on the characteristics of the infusion solution and the exchange of infusion sets are warranted.

An absence of standardized guidelines or protocols in small and medium-sized hospitals might result in nurses not being aware of standard procedures, who may therefore tend to rely on personal experience to guide their practices. Since the level of PIVC nursing knowledge has been identified as an important factor leading to the actual application of PIVC nursing [14, 18], there is a need to provide ongoing customized training and up-to-date standardized protocols to improve the essential knowledge level of nurses in small and medium-sized hospitals regarding PIVC nursing.

The level of PIVC nursing practices was 4.46 ± 0.34 out of 5 points, which was higher than the score of 4.41 ± 0.45 in Choi & Jeong (2020) [14] for nurses in small and medium-sized hospitals. The score in this study was slightly lower than that of 4.66 ± 0.24 found for safety-injection practices by Kim (2021) [21]. This is due to the strengthening of infection control systems and patient safety activities in high-level general hospitals, while small and medium-sized hospitals lack infection control systems, patient safety activities, and support [13]. Therefore, systematic education and monitoring of the effectiveness of education regarding PIVC nursing practices are required for nurses in small to medium-sized hospitals.

The level of the nursing working environment was 2.76 ± 0.47 points out of 4. The mean score in this study was higher than Cho & Kim (2022) [22] study of 2.55 for nurses in small and medium-sized hospitals, and higher than the 2.61 of Kim & Lee (2016) [23] for nurses in high-level general hospitals. The nursing work environment is an organizational characteristic that supports the delivery of professional nursing care. It includes the physical environment that enables nurses to perform their tasks effectively, the human environment that enables interpersonal interactions, and policy aspects. In previous studies, nurses in small and medium sized hospitals tend to have lower nursing work environment perceptions than nurses in advanced general hospitals [22]. Further research on the nursing working environment based on hospital size is needed in the future. The patient safety culture perception was scored 3.73 ± 0.51 out of 5 in this study. This is higher than the mean score of 3.46 for nurses in small and medium-sized hospitals and 3.33 for nurses in large hospitals measured in Cha & Choi’s study [24], which compared patient safety culture awareness among nurses in university and small hospitals. These findings from our study are interpreted to be influenced by the implementation of healthcare accreditation systems and patient safety laws, highlighting the emphasis on patient safety in clinical practice. The only characteristic of the participants that differed significantly with PIVC nursing practices was the length of clinical experience. Participants with 11–15 years of clinical experience had scores for PIVC nursing practices that were higher than for those with < 1 year of clinical experience. This is thought to be due to increased knowledge and performance rate of PIVC nursing practices resulting from the accumulation of clinical experience in practice and education related to infection. Choi & Jeong (2020) [14] found that the level of PIVC nursing practices differed according to the provision of PIVC practice guidelines for preventing infection in the working department. Kim (2021) [21] found differences among nurses at higher-level hospitals according to age and departments. The Survey on the Status of Hospital Nursing Personnel (Hospital Nurses Association, 2022) [25] found that 6–15% of the nurses working in general hospitals and lower-level hospitals had < 1 year of clinical experience. Therefore, a customized education program on repeated PIVC nursing practices is needed for such inexperienced nurses.

This study found that PIVC nursing practices were positively correlated with the practical knowledge of PIVC nursing and patient safety culture perception. This is similar to previous findings that higher levels of knowledge of patient safety during injection practices were associated with a better ability to perform injections [14]. This is consistent with research that perceptions of patient safety culture are positively correlated with patient safety activities [22]. This supports the theory of action that knowledge plays an important role in shaping and governing behavior, and that perceptions of safety culture also have a positive impact on the patient safety activities.

In this study, the factor that had the most significant effect on the PIVC nursing practices was the practical knowledge of PIVC nursing (*β* = 0.43, *p* < .001), followed by the patient safety-culture perception (*β* = 0.29, *p* < .001). Choi and Jeong (2020) [13] found that the organizational culture perception and PIVC practice knowledge influenced the performance of PIVC practices. In a study of factors influencing medical infection-control practices, Yun et al. (2014) [26] also found that higher knowledge, perception and empowerment were associated with a higher level of infection-control practices. Patient safety culture perception is closely related to patient safety during nursing activities since it directly affects safety attitudes and activities. The results of this study are consistent with recent findings of patient safety culture perception being an influential factor for patient safety during nursing activities [22, 27]. Therefore, in order to increase the PIVC nursing practices, it is necessary to change the perception of PIVC nursing practice through periodic education and monitoring at the institutional level, along with efforts to increase nurses’ own knowledge. However, the inherent limitations of small and medium-sized hospitals might result in the institutional education and interventions being less systematic than those in larger hospitals. Therefore, it is necessary to develop policies to promote PIVC nursing practices for patient safety and infection control based specifically on the characteristics of small and medium-sized hospitals.

The nursing working environment was found to not be a significant predictive factor for the level of PIVC nursing practices. This is similar to Cho & Kim (2022) [22] finding that the nursing working environment did not significantly influence patient-safety activities. This may be due to PIVC nursing practices being more influenced by the contextual factors in which PIVCs are applied than by nurses’ perceptions of their working environment [28]. Furthermore, results may vary depending on how the nursing working environment is defined and measured, and it is possible that the tools or indicators used in the present study did not accurately reflect the actual working environment [29]. However, although no significant relationship was found between the nursing working environment and PIVC practices in this study, the working environment was positively related to patient safety-culture perception in the correlation analysis.

The strengths of this study include its focus on PIVC nursing practices based on recently revised and supplemented evidence-based PIVC guidelines, and that it identified the influencing factors of PIVC nursing practices among nurses in small and medium-sized hospitals to provide a basis for nursing practice to improve the quality of nursing care in small and medium-sized hospitals. However, the generalizability of the study findings might have been reduced by the use of a convenience sample of nurses in small and medium-sized hospitals in only certain regions. In addition, the KR-20 of the tool used to measure PIVC nursing knowledge was found to be relatively low. A KR-20 value of 0.41 indicates that the internal consistency is moderate, meaning that the items in the test have some consistency, but the reliability of the test results is moderate. This indicates that the test may have some limitations when it comes to measuring that particular construct [30]. The KR-20 is a statistical measure used to estimate the reliability or internal consistency of a test, particularly for tests with dichotomous (yes/no) items. Higher values of the KR-20 coefficient indicate greater internal consistency reliability, suggesting that the items on the test are measuring the same underlying construct consistently [30]. The tool of PIVC nursing knowledge is to investigate the level of knowledge about preparing and administering medications, following aseptic technique, assessing phlebitis, and more. The KR-20 score was somewhat low because the tool measured the nurse’s overall knowledge of peripheral IV practice rather than some concept. Furthermore, the use of self-reported rather than observed measures of PIVC nursing practices may need to be improved in future studies. PIVC nursing practices in inpatient nursing is a frequent and important nursing activity for infection control and patient safety.

Future studies are needed to identify educational needs based on the results of this study and to develop educational and intervention programs aimed at improving PIVC nursing practices in small and medium-sized hospitals and to evaluate their effectiveness.

## Conclusion

It was found that PIVC practice knowledge and patient safety culture perception were significant factors affecting the PIVC nursing practices of nurses in small and medium-sized hospitals. To improve the PIVC nursing practices, it is necessary to provide education and training based on the latest standard or guideline to acquire knowledge and skills, and to establish and support an institutional safety culture.

This study is significant in that it examined the factors that influence PIVC nursing practice in small and medium-sized hospitals, which play a pivotal role in the healthcare services in South Korea, but tend to lack systems for patient safety. Based on the findings of this study, we make the following recommendations: First, it is necessary to develop a standardized PIVC training program based on latest guideline in small and medium-sized hospitals to improve the uniformity and level of practical knowledge and skills. Second, campaigns and programs tailored to the conditions of small and mid-sized hospitals to strengthen patient safety culture perception should be implemented to ensure that a culture of safety. Third, since self-reported surveys have lower objectivity, it is necessary to introduce and develop objective tools or methodologies for assessing the actual ability to perform PIVC practices. This would help to close the gap between actual skills and self-reported outcomes, and provide a more-accurate picture of the effectiveness of education and training.

## Data Availability

The dataset used or analyzed during the current study are available from the corresponding author on reasonable request.

## References

[1] Nickel B (2019). Peripheral intravenous access: applying infusion therapy standards of practice to improve patient safety. Crit Care Nure.

[2] Kim JI, Lee JH, Chang OJ (2013). Perceived importance and performance of intravenous fluid therapy by nurses in small-medium general hospitals. J Korean Acad Fundam Nurs.

[3] López JLZ, Vilela AA, Palacio EF, Corral JO, Martí CB, Portal PH (2014). Indwell times, complications and costs of open vs closed safety peripheral intravenous catheters: a randomized study. J Hosp Infect.

[4] Korea Hospital Association (2017). National Hospital Status.

[5] Higgins N, Keog S, Rickard C (2015). Evaluation of a pilot educational program on safe and effective insertion and management of peripheral intravenous catheters. JAVA.

[6] Keleekai NL, Schuster CA, Murray CL, King MA, Stahl BR, Labrozzi LJ (2016). Improving nurses’ peripheral intravenous catheter insertion knowledge, confidence, and skills using a Simulation-based blended Learning Program. J Simul Healthc.

[7] Paquet F, Boucher LM, Valenti D, Lindsay R (2017). Impact of arm selection on the incidence of PICC complications: results of a ran-domized controlled trial. J Vasc Access.

[8] Ullman AJ, Long DA, Rickard CM (2013). Prevention of central venous catheter infections: a survey of paediatric ICU nurses’ knowledge and practice. Nurse Educ Today.

[9] Vizcarra C, Cassutt C, Corbitt N, Richardson D, Runde D, Stafford K (2014). Recommendations for improving safety practices with short peripheral catheters. J Infus Nurs.

[10] Moon JE, Jang KS (2018). The performance of healthcare-associated infection control guideline among hospital nurses: a structural equation model. Iran J Public Health.

[11] Kim HH. The effects of perceived organizational culture for infection control and self-efficacy on the standard precautions of emergency room nurses [master’s thesis]. Cheongju: Chungbuk National University. 2018:1–70.

[12] Korea Centers for Disease Control and Prevention (KCDC). 2016. Operation of the nationwide surveillance system for healthcare-associated infection in intensive care units. Cheongju: KCDC; 2016 December. Report No.: 11-1352159-000636-.

[13] The Hospital Nurses Association. Hospital Nursing Staffing Survey 2021. 2022 February. https://khna.or.kr/home/pds/utilities.php.

[14] Choi SA, Jeong SY (2020). Factors influencing compliance in intravenous practice for infection prevention among nurses in small and medium hospitals. Korean Acad Fundam Nurs.

[15] Lake ET (2007). The nursing practice environment. Med Care Res Rev.

[16] Cho EH, Choi M, Kim EY, Yoo IY, Lee NJ (2011). Construct validity and reliability of the Korean version of the practice environment scale of nursing work index for Korean nurses. J Korean Acad Nurs.

[17] Kim JE, Kang MA, An KJ, Sung YH (2007). A survey of nurses’ perception of patient safety related to hospital culture and reports of medical errors. J Korean Clin Nurs Res.

[18] Song KJ. Factors that affect safe injection practices of nurses at small and medium sized hospitals: application of theory of planned behavior[master’s thesis]. Konyang University. 2020 Feb.

[19] Hospital Nurses Association. Evidence-based clinical nursing practice guideline 2017. [cited 2023 March 6]. https://khna.or.kr/home/data/230223/khna_guide_2023_1.pdf.

[20] Ministry of Health and Welfare. Safety Guidelines for Injection Infection Prevention. 2021.

[21] Kim YM. Effects of professionalism and self-leadership of nurses in tertiary hospital on safe injection practices[master’s thesis]. Konyang University. 2021.

[22] Cho YO, Kim MS (2022). Effects of perceived safety culture, nursing work environment, and professional self-concept on patient safety care activity of nurses in small-medium sized hospitals. J Korean Soc Health Inf Stat.

[23] Kim J, Lee TW (2016). The influence of nursing practice environment, compassion fatigue and compassion satisfaction on burnout among clinical nurses. J Korean Clin Nurs Res.

[24] Cha BK, Choi J (2015). A comparative study on perception of patient safety culture and safety care activities: comparing university hospital nurses and small hospital nurses. J Korean Acad Nurs Adm.

[25] Hospital Nurses Association. Nursing personnel policy and prospect of hospital nursing 2022. [cited 2023 March 6]. https://khna.or.kr/home/pds/utilities.php.

[26] Yun JY, Kim SO, Kim IS (2014). Influencing factors on practice of healthcare-associated infection control among clinical nurses. Korean J Occup Health Nurs.

[27] Han IJ, Han YR (2023). Mediating effects of job satisfaction between nurses’ perceptions of patient safety culture and their safety nursing activities. J Korean Acad Fundam Nurs.

[28] Pugliese G, Gosnell C, Bartley JM, Robinson S (2010). Injection practices among clinicians in United States health care settings. Am J Infect Control.

[29] Aiken LH, Clarke SP, Sloane DM, Lake ET, Cheney T (2008). Effects of hospital care environment on patient mortality and nurse outcomes. J Nurs Adm.

[30] Kaplan RM, Saccuzzo DP (2012). Psychological testing: principles, applications, and issues.

